# Sensitive and rapid detection of *Ortleppascaris sinensis* (Nematoda: Ascaridoidea) by loop-mediated isothermal amplification

**DOI:** 10.7717/peerj.7607

**Published:** 2019-09-06

**Authors:** Jinhong Zhao, Wei Xu, Genjun Tu, Yongkang Zhou, Xiaobing Wu

**Affiliations:** 1 Department of Parasitology, Wannan Medical College, Wuhu, Anhui, China; 2 Provincial Laboratory of Conservation and Exploitation of Biological Resources, College of Life Sciences, Anhui Normal University, Wuhu, Anhui, China; 3 The National Nature Reserve of Chinese Alligator in Anhui, Xuanzhou, Anhui, China

**Keywords:** *Ortleppascaris sinensis*, Diagnosis, Loop-mediated isothermal amplification, Rapid detection, The Chinese alligator

## Abstract

*Ortleppascaris sinensis* is the dominant nematode species infecting the gastrointestinal tract of the captive Chinese alligator, a critically endangered species. Gastrointestinal nematode infection may cause a loss of appetite, growth, a development disorder, and even mortality in alligators, especially young ones. This research first establishment a loop-mediated isothermal amplification (LAMP) assay in rapidly identifying *O. sinensis*, upon the basis of the complete internal transcribed spacers (ITS) gene. Eight sets of primers were designed for recognition of the unique conserved ITS gene sequences, and one set was selected to be the most suitable primer for rapid detection. The specific as well as the sensitive features of the most appropriate primer in LAMP reactions for *O. sinensis*, and feces specimens of Chinese alligators suffering from *O. sinensis* were determined. Turbidity monitoring and Te Visual Reagent methods were used for determining negative and positive consequences. According to this study, amplification and visualization of the target DNA could be realized through two detection approaches during 50 min at 65 °C isothermal temperature. The sensitivity of LAMP was a detecting limitation of 3.46 pg/µl DNA. No cross-reactions were found between *O. sinensis* and any other of the nine heterologous nematode parasites, which shows the outstanding specific features of the primers. The LAMP assay could also perform a detection of target DNA of *O. sinensis* in the feces samples of Chinese alligators. This LAMP assay is useful for directly detecting *O. sinensis* in the Chinese alligator breeding centers, particularly due to its rapidity, simplicity and low cost.

## Introduction

*Ortleppascaris sinensis* is the dominant species infecting the gastrointestinal tract of captive Chinese alligators in the National Nature Reserve of Chinese Alligators (NNRCA) (Zhao et al., 2016). The presence of the nematode *O. sinensis* can cause superficial ulcers and granulomatous inflammation in the entire gastric walls of infected Chinese alligators (Zhao et al., 2015). Chinese alligator in the NNRCA (especially young ones) infected with nematodes in the gastrointestinal tract show a decline in appetite, and even mortality (Wang, Nie & Wang, 1995). Although crocodiles are very strong, gastrointestinal nematode infections also may be occasionally associated with disease. There are reports that infection with *Dujardinascaris* is related to gastric ulceration and runting in hatchlings (Huchzermeyer, 2003; Ladds & Sims, 1990).

The adult *O. sinensis* can be found in the stomach and intestine of Chinese alligators. The Chinese alligator *Alligator sinensis* Fauvel 1879 (Crocodilian: Alligatoridae) is a critically endangered species native to eastern China. The wild alligator population is only around 120–150 at present (Zhao et al., 2018). The NNRCA is the largest alligator facility in China, housing 15,000 individuals. Although it is not permitted to dissect wild or captive Chinese alligators for parasite investigation on a large scale, the eggs of *O. sinensis* can be inspected via the conventional method of a physiological saline direct smear and natural sedimentation of the feces of the Chinese alligator. It is well-known that the conventional scatology methods to acquire the eggs of parasites take time and effort, and have a low sensitivity (Li et al., 2012). Molecular biological approaches, including traditional PCR, multiplex PCR or real-time PCR can also be used for the detection of Ascaridae parasites (Cuttell et al., 2012). For example, traditional PCR was used to amplify the gene of *O. sinensis* to identify the species (Zhao et al., 2016). PCR assays appear promising for rapid, sensitive and specific diagnosis. However, the PCR assays require the use of special and expensive instruments and consumables, and thus can not be used for on-site detection (Mwangi et al., 2018). In addition, the presence of inhibitors in crude biological specimens does not allow the activation of the Taq DNA polymerase utilized in PCR assays.

The newly invented loop-mediated isothermal amplification (LAMP) approach only requires a water bath controlled by temperature. It is based upon auto cycling strand displacement DNA synthesis with the existence of Bst DNA polymerase in isothermal circumstances for 1 h (Notomi et al., 2000). Rapidity and simplicity in operation are the outstanding features of LAMP, which merely requires a thermostatic environment (ranging from 60 to 68 °C) of less than 40 min for amplification (Ting & Kun, 2018; Wang et al., 2016). These characteristics fit in well with the epidemiological investigation and on-site detection especially when experimental settings are limited (Niu et al., 2018; Hirayama et al., 2006). Until now, LAMP assay has been regarded as an excellent diagnosis approach in detecting parasites, including *Schistosoma japonicum* (Xu et al., 2010), *Meloidogyne* (Peng et al., 2017), *Trichinella spiralis* (Li et al., 2012), *Plasmodium falciparum* (Kersting et al., 2014), *Giardia* (Crannell et al., 2015), *Leishmania donovani* (Mondal et al., 2016) and so on. The application of LAMP assay in diagnosing parasitic diseases have also been reported in recent years, such as toxoplasmosis (Sotiriadou & Karanis, 2008), angiostrongylosis (Liu et al., 2011), and cryptosporidiosis (Crannell et al., 2014).

Considering the dangers of *O. sinensis* nematodes to the health of captive Chinese alligators, as well as the underlying economic influence of endangered species, the LAMP assay was used to evaluate the sensitivity, specificity and clinical application of *O. sinensis* based the internal transcribed spacers (ITS) gene. This LAMP assay is a valuable diagnostic tool for the direct detection of *O. sinensis* in Chinese alligators in breeding centers, particularly due to the speed of the test, simplicity, and low cost.

## Materials and Methods

### Parasites, fecal samples and genomic template preparation

The *O. sinensis* used in the study were obtained from the Chinese alligator, *Alligator sinensis*, in the NNRCA in Anhui (30°94′N, 118°79′E), a significant breeding center in southern China that plays a crucial role in the preservation of *Alligator sinensis* (Zhao et al., 2014). Adult nematodes *O. sinensis* were obtained from the stomach and intestine of the *Alligator sinensis*, washed in physiological saline, identified to species *O. sinensis* and fixed in 75% ethanol (Zhao et al., 2016). The nematodes, *Ascaris lumbricoides*, *Anisakis* sp., *Trichinella spiralis*, *Cucullanus elongatus*, *Taenia solium*, *Taenia asiatica*, *Ligula* sp., *Fasciola gigantica*, and *S. japonicum* were obtained from the Department of Parasitology, Wannan Medical College, and several other universities (Table 1). A DNeasy Blood & Tissue Kit (Tiangen Biotech Beijing Co., Ltd., Beijing, China) was used, following manufacturer instructions, to extract genomic DNA from these parasites. Stool samples were also collected from the NNRCA in Anhui from June 2016 to May 2019. All of the fecal samples were preserved at −80 °C for 1 week to kill the eggs after collection, and the extraction of genomic DNA from the stool samples was conducted by the Faeces Kit (Tiangen Biotech Beijing Co., Ltd., Beijing, China). All these DNA samples were preserved at −20 °C prior to subsequent analysis.

**Table 1:** Parasites used in this study.

Species	Source
*Ortleppascaris sinensis* (OS)	Department of Parasitology, Wannan Medical College, Wuhu, China
*Ascaris lumbricoides* (AL)	Department of Parasitology, Wannan Medical College, Wuhu, China
*Anisakis* sp. (AN)	Department of Ecology, Evolution and Marine Biology, University of California, Santa Barbara, USA
*Trichinella spiralis* (TSP)	Department of Parasitology, School of Basic Medical Sciences, Zhengzhou University, Zhengzhou, China
*Cucullanus elongatus* (CE)	Department of Ecology, Evolution and Marine Biology, University of California, Santa Barbara, USA
*Taenia solium* (TS)	Department of Parasitology, School of Basic Medical Sciences, Dali University, Dali, China
*Taenia asiatica* (TA)	Department of Parasitology, School of Basic Medical Sciences, Dali University, Dali, China
*Ligula* sp. (LI)	Department of Parasitology, Wannan Medical College, Wuhu, China
*Fasciola gigantica* (FG)	Department of Parasitology, Wannan Medical College, Wuhu, China
*Schistosoma japonicum* (SJ)	Department of Parasitology, Wannan Medical College, Wuhu, China

### Design of LAMP primers

To design the ITS-specific LAMP primers, ITS sequences of *O. sinensis* were downloaded from the NCBI GenBank database: KM891739 (Zhao et al., 2016). The Primer Explorer V4 software (http://primerexplorer.jp/lamp) was utilized for analysis of the sequence. For acquisition of the best suitable primers, eight sets of primers were designed, consisting of outer forward primers (F3), outer backward primers (B3), inner forward primers (FIP), inner backward primers (BIP) and two loop primers (LF and LB). These loop primers were designed for acceleration of the LAMP amplification reaction. Traditional PCR was carried out with the ITS-F3 and ITS-B3 primer pairs so as to make a validation and internal control for sensitive and specific features of LAMP assay. Each primer was synthesized in a commercial manner (Beijing TsingKe Biotechnology Co., Ltd., Beijing, China).

### LAMP reaction

The LAMP reaction was conducted in a 25 µl reaction system (LAMP DNA Amplification Kit; Eiken Chemical Co., Ltd., Tochigi, Japan) including below reagents (eventual concentration): 10 mM KCl, 10 mM (NH_4_)_2_SO_4_, 20 mM Tris–HCl (pH 8.8), 0.1% Tween 20, 0.8M betaine, eight mM MgSO_4_, 1.4 mM each dNTP, 8 U Bst DNA polymerase and 10 pmol for F3 and B3, 20 pmol for LF and LB and 40 pmol for FIP and BIP. Eventually, one μl template genomic DNA was added to the reaction tube (Eiken Chemical Co., Ltd., Tochigi, Japan). Distilled water was used as the negative control. The reaction was performed at 60–67 °C for 50 min at 1 °C intervals, respectively. Followed by 80 °C for 5 min to terminate the amplification within a dry bath incubator (OSE-DB-02; Tiangen Biotech Beijing Co., Ltd., Beijing, China).

### Inspection of LAMP products

For direct visual inspection, one µl of Te Visual Reagent (TVR) (Tianjin JieYiTe Technology Co., Ltd., Tianjin, China) was added to the 25 µl reaction system preceding LAMP reactions (Niu et al., 2018). The LAMP product changed in color from colorless to green for a positive reaction, while the color did not become green and maintained colorless in the negative reaction. The color change is visible with the naked eye under natural light without additional instruments. To monitor the turbidity (Mori et al., 2001), spectrophotometric analysis was used to conduct a real-time LAMP amplification through records of absorbance at 400 nm each for 6 s under a Loopamp real-time turbidimeter (LA-230; Eiken Chemical Co., Ltd., Tochigi, Japan). Standardization of reaction time under the inspection of the Loopamp real-time turbidimeter is according to the appearance of the positive curve.

### PCR detection

The PCR reaction was conducted in a 25 µl reaction mixture, which included 12.5 µl PCR master mixed reagents (Takara Biotechnology Co., Ltd., Beijing, China), 10 µmol/l ITS-F3 and ITS-B3 primers as well as one µl DNA template. Including outer primers, the PCR product size should be 209 bp. This initial reaction was performed at 94 °C for 1 min, with subsequent 35 cycles at 94 °C for 30 s, 56 °C for 5 min. The eventual expansion step was conducted at 72 °C for 10 min. Analysis of such PCR product was carried out with utilization of 2% agarose gel electrophoresis and documented under a Bio-Rad Gel Doc EQ imaging system (Bio-Rad, Hercules, CA, USA) for semi-quantity analyzation.

## Results

### Primers of the LAMP reaction

**Figure 1:**
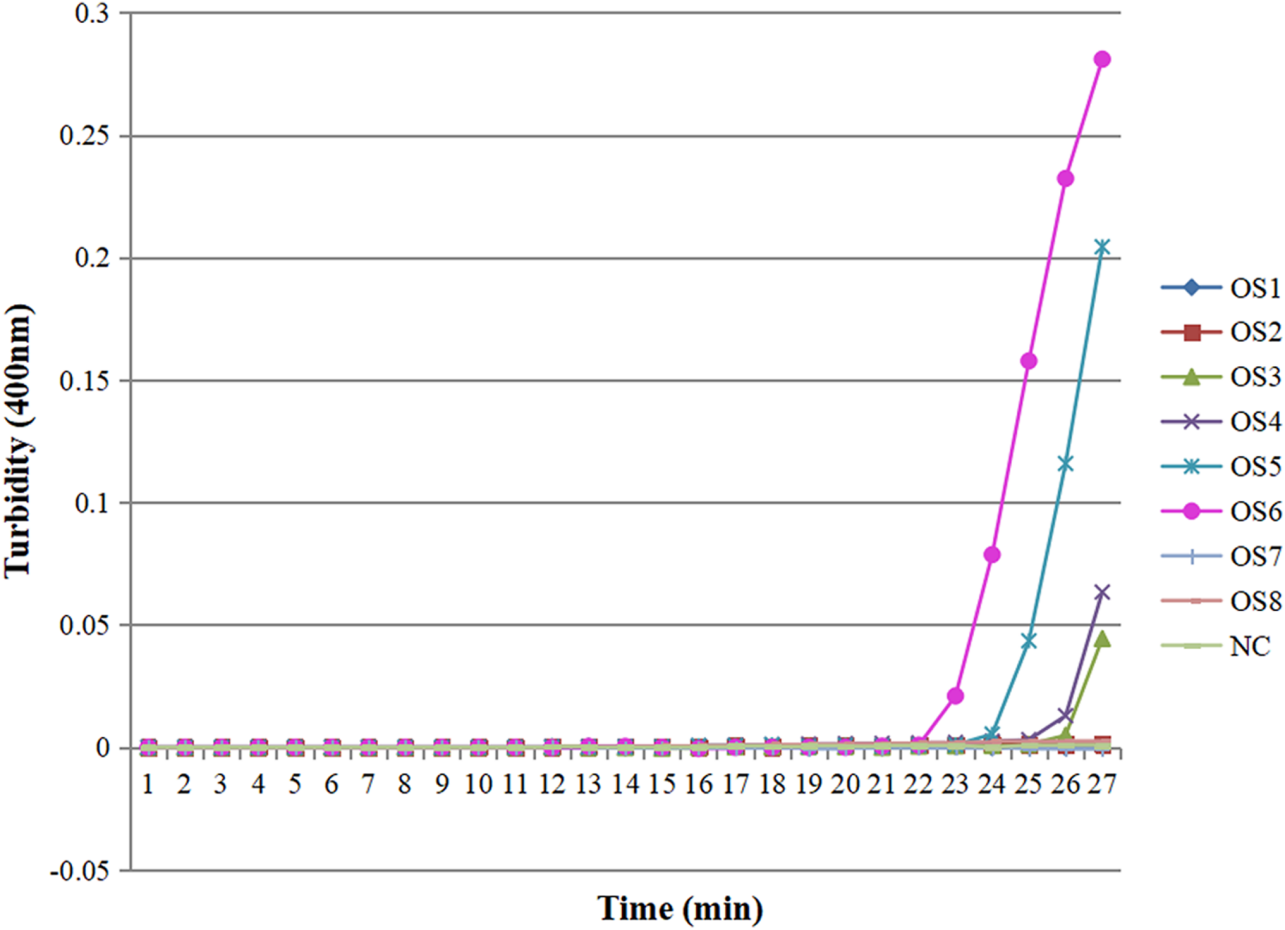
Eight sets of primers of the LAMP reaction for detection of *O. sinensis*. Turbidity was monitored by a Loopamp realtime turbidimeter at 400 nm every 6 s, the curve graph was analyzed every 1 min. NC means double-distilled water.

Eight sets of LAMP primers were designed based on the ITS gene sequence for the detection of *O. sinensis*. Under these same real-time LAMP reaction conditions, four curves occurred after a 27 min LAMP reaction, which demonstrated the successful amplification of the target sequence by these four sets of primers. Among them, the OS6 was able to amplify the target gene with the most rapid speed, and was considered the fastest optimized reaction primer (Fig. 1). Therefore, the OS6 primers were selected to be the eventual set of LAMP primers in detecting *O. sinensis* (Table 2).

**Table 2:** Sequences of the ITS primer set used for specific detection of *Ortleppascaris sinensis*.

Primer	Type	Sequence (5′ to 3′)
OS6-F3	Forward outer	GCAGACACATTGAGCACT
OS6-B3	Backward outer	GGAGCTCGATAACGAAAGC
OS6-FIP	Forward inner	ACGACCCTCAGCCAGACGTGAAGACTTTGAACGCGCATTG
OS6-BIP	Backward inner	GGCGTCATCGCGTTGATACGTCTGAGCGTAGTATCCTGAA
OS6-LF	Loop forward	AACGGGAAAGAACCCGAT
OS6-LB	Loop backward	CGTGCTATCAGAAATGCAAGT

### Temperature of the LAMP reaction

**Figure 2:**
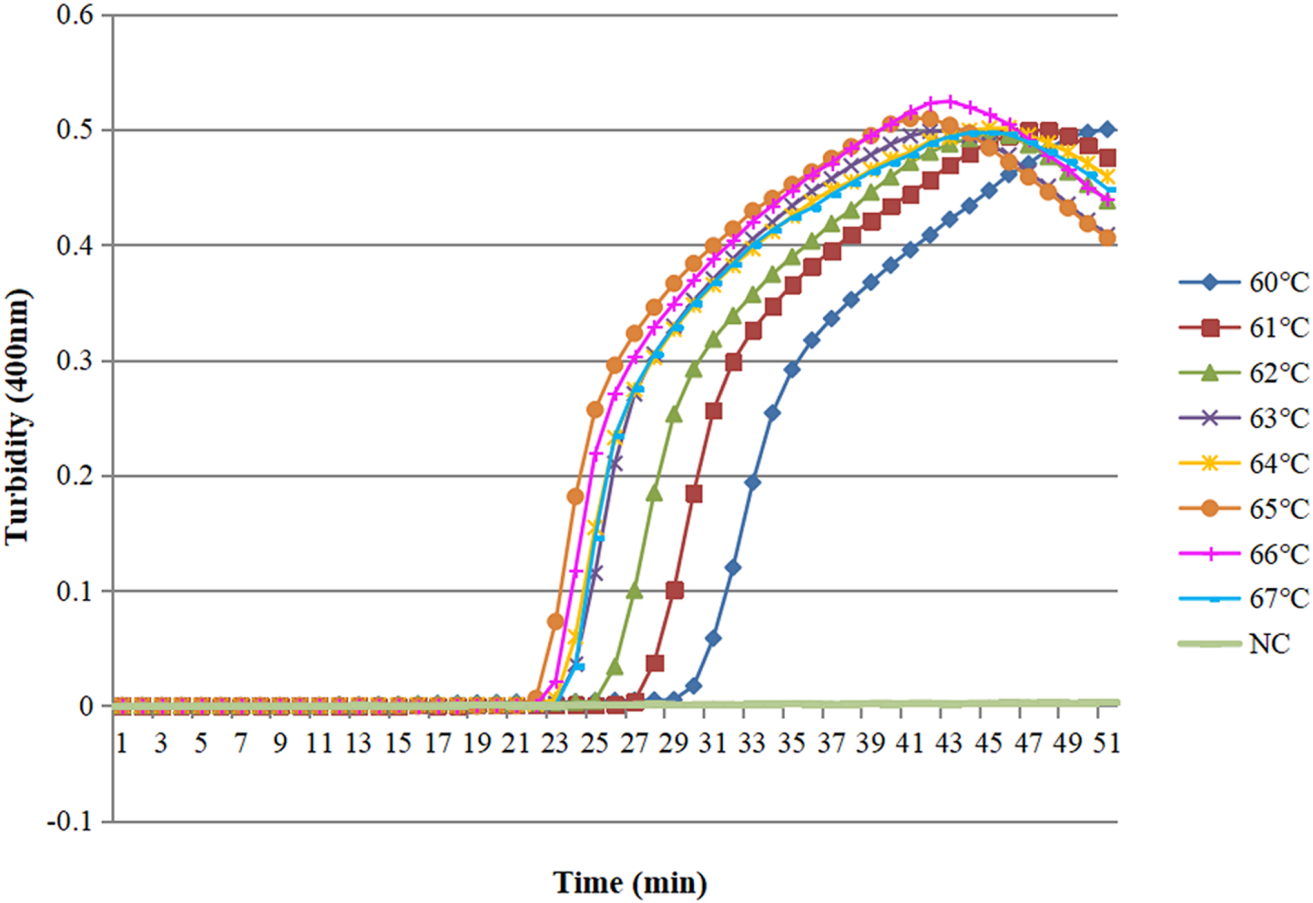
Different temperatures of the LAMP reaction for detection of *O. sinensis*. Turbidity was monitored by a Loopamp real-time turbidimeter at 400 nm every 6 s, the curve graph was analyzed every 1 min. Eight kinetic curves were generated at various temperatures (60–67 °C, 1 °C intervals) with target pathogens DNA at the level of 0.346 ng per reaction. The curves from 63 °C to 67 °C showed robust amplification. A total of 65 °C is the first to occur the graphs. NC means double-distilled water.

For optimization of the reaction temperature of the real-time LAMP in detection of *O. sinensis* based on the ITS gene, different temperatures were executed ranging between 60 and 67 °C at 1 °C intervals. We found that the graphs from 63 to 67 °C are the better suitable reaction temperature ranges, showed robust amplification within 50 min under the inspection of the Loopamp real-time turbidimeter. However, the turbidity of 65 °C occurred earlier than the other temperatures with amplification products being relatively higher (Fig. 2). Thus, 65 °C was chosen as the final reaction temperature of the real-time LAMP.

### Specificity of LAMP assays

**Figure 3:**
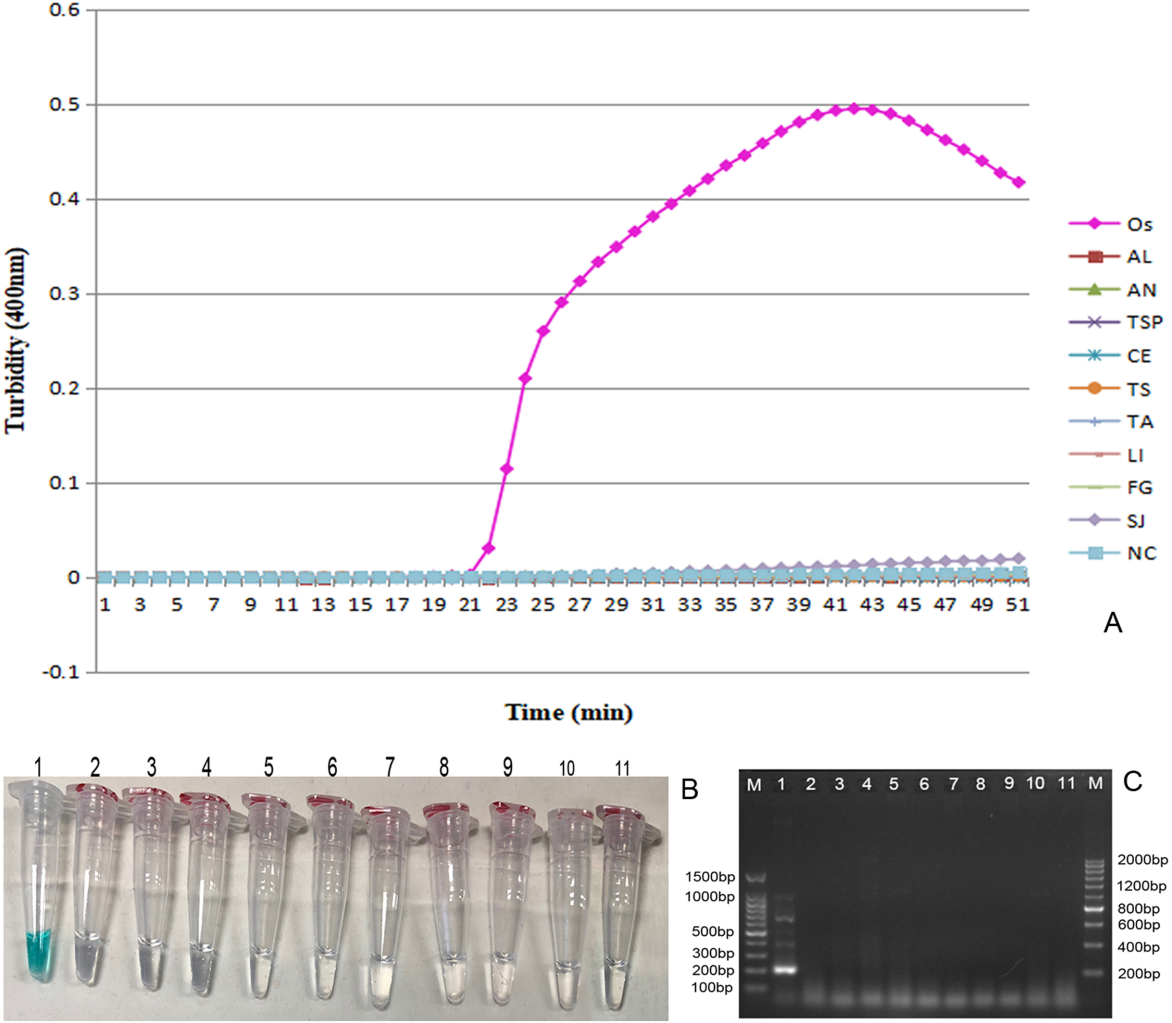
The specificity of the LAMP reaction for detection of *O. sinensi*. (A) Turbidity was monitored by a Loopamp real-time turbidimeter at 400 nm every 6 s, the curve graph was analyzed every 1 min. (B) A TVR reagent detection method was executed. One µl TVR reagent was added to 25 µl LAMP reaction mixture before the LAMP reaction. Amplification was performed at 65 °C for 50 min. (C) PCR products were analyzed by 2% agarose gel electrophoresis and stained with ethidium bromide. Tubes and lanes: (1) OS (*Ortleppascaris sinensi*); (2) AL (*Ascaris lumbricoides*); (3) AN (*Anisakis* sp.); (4) TSP (*Trichinella spiralis*); (5) CE (*Cucullanus elongatus*); (6) TS (*Taenia solium*); (7) TA (*Taenia asiatica*); (8) LI (*Ligula* sp.); (9) FG (*Fasciola gigantica*); (10) SJ (*Schistosoma japonicum*); (11) NC (double-distilled water).

To verify the specificity of LAMP assays for *O. sinensis* based on ITS gene, we used *O. sinensis* as the positive control group. Several heterologous parasites including *Ascaris lumbricoides*, *Anisakis* sp., *Trichinella spiralis*, *C. elongatus*, *Taenia solium*, *Taenia asiatica*, *Ligula* sp., *F. gigantica* and *S. japonicum*, in addition to double distilled water were selected to be the negative control groups. Two methods, TVR reagent color detection and Loopamp real-time turbidity detection, were utilized for detection of LAMP products. As shown in Fig. 3A, the growing turbidity curve presented within 50 min only when *O. sinensis* was used as the template, and not when the other nine heterologous parasites and the distilled water were used. Such outcomes indicate that the OS6 primer possessed specificity for LAMP identification of *O. sinensis*. Before LAMP reactions, one µl TVR identification reagent was added into a 25 µl LAMP reaction mixture. All those positive reaction samples turned green (Fig. 3B, tube 1); in contrast, the negative reaction samples remained colorless (Fig. 3B, tubes 2–11). The results of visual color detection are the same as the results using the real-time turbidity detection method. PCR using the OS6-F3 and OS6-B3 primer pair were also performed. The target band (209 bp) only appeared in the *O. sinensis* positive control group (Fig. 3C).

### Sensitivity of LAMP assays

**Figure 4:**
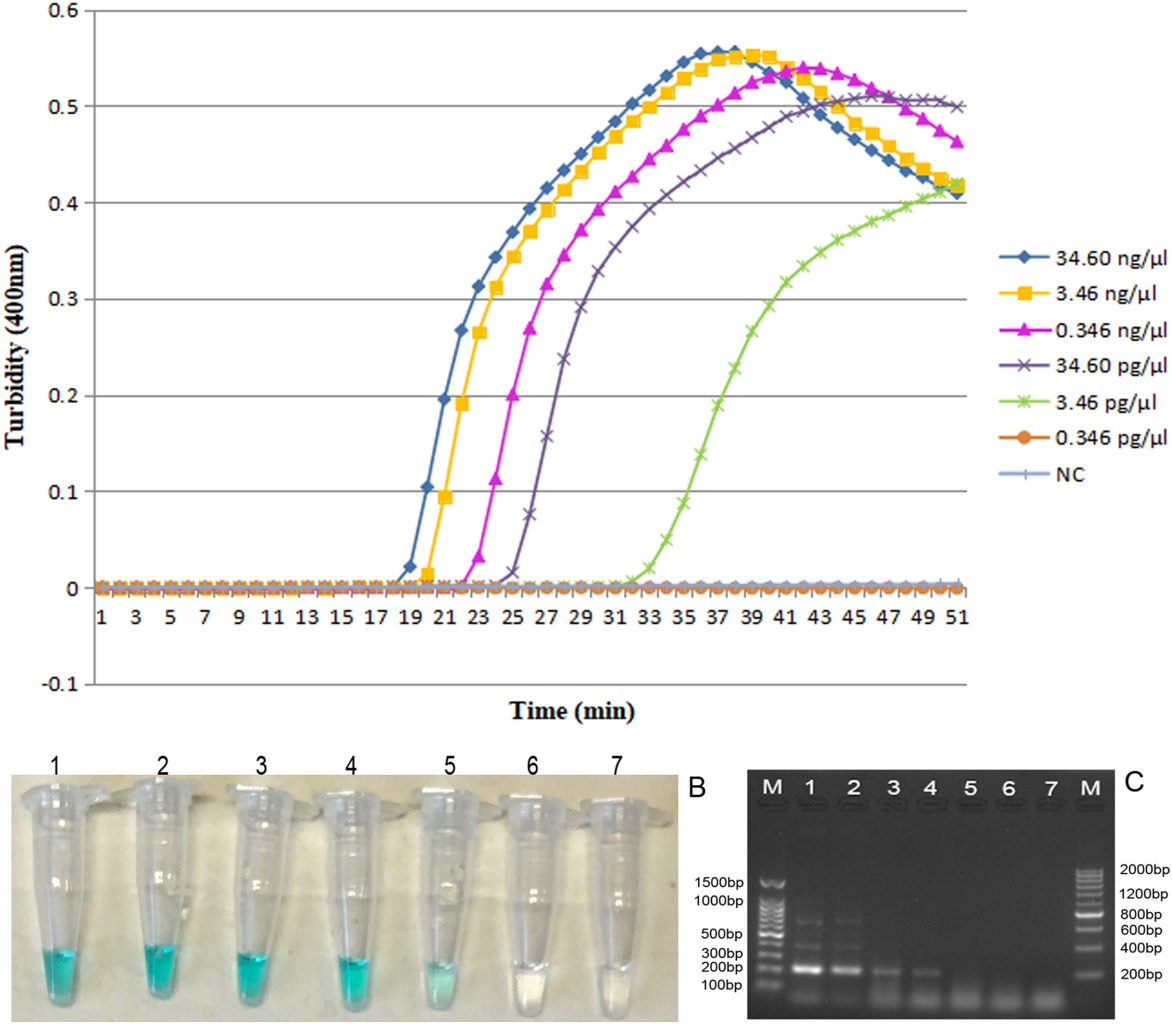
The sensitivity of the LAMP reaction for detection of *O. sinensis*. (A) Turbidity was monitored by a Loopamp real time turbidimeter at 400 nm every 6 s, the curve graph was analyzed every 1 min; (B) The TVR visual color detection was compared using the addition of one µl TVR reagent to 25 µl LAMP reaction mixture before the LAMP reaction; (C) PCR products were analyzed by 2% agarose gel electrophoresis and stained with ethidium bromide. Tubes and lanes: (1) 34.60 ng/µl; (2) 3.46 ng/µl; (3) 0.346 ng/µl; (4) 34.60 pg/µl; (5) 3.46 pg/µl; (6) 0.346 pg/µl; (7) NC (double-distilled water).

To determine sensitivity of LAMP assays for *O. sinensis* based on the ITS gene, the DNA was extracted from *O. sinensis* and the purified DNA template was diluted by a series of 10-fold. According to the turbidity graph of Loopamp real-time turbidimeter, the positive curve can appear on the screen within 50 min by concentration ranges of between 34.60 ng/µl and 0.346 pg/µl (Fig. 4A). So the detecting limitation of LAMP assays for the DNA concentration of *O. sinensis* was 3.46 pg/µl. Furthermore, we monitored the LAMP results utilizing a direct TVR visual approach. Before the LAMP reaction, one µl TVR detection reagent was added to a 25 µl reaction mixture. Every positive reaction sample became green, while the negative reaction samples remained colorless. The concentration range of positive reactions was between 3.46 and 0.346 pg/µl (Fig. 4B, tubes 1–6). These results suggest the similar sensitivity of the two detection approaches. PCR amplification utilizing the OS6-F3 and OS6-B3 primer pair was also conducted for validation and internal control. According to the results, the detection limit of the PCR attained 34.6 pg/µl (Fig. 4C).

### LAMP detection of *O. sinensis* in fecal samples

**Figure 5:**
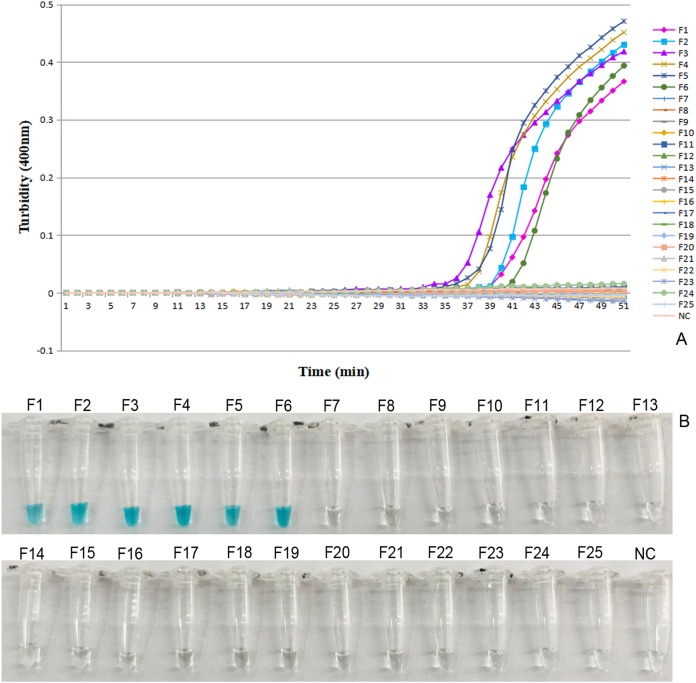
Detection of fecal samples of the LAMP reaction for *O. sinensis*. (A) Turbidity was monitored by a Loopamp real time turbidimeter at 400 nm every 6 s, the curve graph was analyzed every 1 min; (B) The TVR visual color detection was compared using the addition of one µl TVR reagent to 25 µl LAMP reaction mixture before the LAMP reaction. Tubes: F1–F6 was the fecal samples of the Chinese alligator infected with the *O. sinensis* and F7–F25 was the fecal samples uninfected Controls; NC means double-distilled water.

Altogether, 25 fecal specimens consisting of six positive and 19 negative fecal samples were prepared for LAMP assays. All six positive fecal samples were previously identified by founding adult nematodes *O. sinensis* in the stomach and intestine of the *Alligator sinensis*, and PCR also obtained positive amplification for the ITS gene. Furthermore we also found the eggs of *O. sinensis* in the positive fecal samples. These methods were conducted according to Zhao et al. (2016). However, all of these results of the above methods which were negative were regarded as negative fecal samples. The results of the *O. sinensis* by LAMP assay were in complete agreement with those we have identified before. We observed that only six curves appeared on the screen for the positive fecal samples within 50 min which were infected with *O. sinensis* according to the results of Loopamp real-time turbidimeter (Fig. 5A). No curves appeared on the screen for the 19 negative fecal samples which were not infected with *O. sinensis*. Identical outcomes have been found for color visualization assays (Fig. 5B). This result shows that *O. sinensis* infection in the fecal samples of Chinese alligators was detectable with adoption of LAMP assay.

## Discussion

The nematode *O. sinensis* was the dominant species infecting the gastrointestinal tract of the captive Chinese alligator in China (Zhao et al., 2016). There have been reports that the nematode *O. sinensis* can cause superficial ulcers in the mucous layer and granulomatous inflammation in the submucous layer on the entire gastric walls of the *Alligator sinensis* infected with nematode *O. sinensis* (Zhao et al., 2015). *Ortleppascaris* adults have been found in the stomachs and intestines of alligators and crocodiles (Tellez, 2013; Waddle et al., 2009; Zhao et al., 2016), and larvae have been found in the livers of amphibians (Moravce, 1995; e Silva et al., 2013) and the mesenteries and the body cavities of fishes and frogs infected in a natural manner in Africa and the USA (Huchzermeyer, 2003; Tellez, 2013). Fish, frogs and amphibians are the main foods consumed by crocodiles and alligators, which greatly increases the risk of nematode *Ortleppascaris* for crocodiles and alligators. The Chinese alligator is a critically endangered species native to eastern China, therefore early and rapid diagnosis of the parasite in the fecal samples of the Chinese alligator will help control the infection of parasites which will help to protect the endangered species.

Currently, this LAMP approach has been utilized to rapidly diagnose protozoa, fluke, tapeworm and nematodes in clinical molecular diagnosis of parasitic diseases (Li et al., 2013; Soliman & El-Matbouli, 2006). As far as we know, rapid detection of *O. sinensis* by LAMP assay have not been reported. This research is intended to establish and optimize a real-time LAMP assay to detect the nematodes *O. sinensis* based on the ITS gene, for development of a sensitive, rapid and reliable test so as to diagnose and control the nematode *O. sinensis* earlier. The ITS gene is effective for identifying nematodes and for researching genetic variations (Marucci et al., 2013; Zhao et al., 2016).

In this study, a real-time turbidimeter and colorimetric indicator were used to inspect the LAMP products. The positive curve monitored under a Loopamp real-time turbidimer can be seen directly through the screen during the real-time LAMP (Monica, Rathinasabapathi & Ramya, 2019; Liu et al., 2012). Furthermore, the presence of loop primers can increase the efficiency of amplification and make the detection more rapidly. Even DNA pathogens with low levels can be detected by real-time LAMP (Cai et al., 2018). When the sensitivity of the LAMP assay for *O. sinensis* detection was evaluated, the detection limit of the *O. sinensis* by the LAMP assay was 3.46 pg/µl, and there were no cross reactivities found with the DNA of any of the other nine heterologous nematode parasites (*Ascaris lumbricoides*, *Anisakis* sp., *Trichinella spiralis*, *C. elongatus*, *Taenia solium*, *Taenia asiatica*, *Ligula* sp., *F. gigantica* and *S. japonicum*) in this study. Moreover, we evaluated the practical applicability of LAMP assay for *O. sinensis* in 25 fecal samples from the Chinese alligator. All of the positive samples for the *O. sinensis* were successfully identified with the LAMP assay. The LAMP detection of *O. sinensis* could be completed within 50 min at 65 °C. Traditional scatology methods and normal PCR require much time and are dependant on special equipment. There were 2–2.5 pairs of sequences working on the target sequence with the LAMP method, making it more rapid and sensitive than the normal PCR method (Nakano et al., 2015). On the other hand, the color visualization detecting approach greatly enlarges the scope of LAMP assays. A water bath or any other heating device that can heat under the temperature 65 °C for 1 h, is adequate to detect *O. sinensis*. Moreover, the LAMP assay is easy to operate. It performs under isothermal conditions and can even be used by persons with no operating experience.

## Conclusion

We established a specific, sensitive and rapid LAMP assay based on the ITS gene in detecting the nematode *O. sinensis* that infects the Chinese alligator. DNA templates of the *O. sinensis* and the non *O. sinensis* were successfully recognized with the LAMP assay. The detection limit of this assay is 3.46 pg/µl which can be recognized within 50 min of isothermal reaction at 65 °C. The LAMP assay could efficiently identify *O. sinensis* in positive fecal samples. The nematode *O. sinensis* was the dominant species infecting the gastrointestinal tract of the Chinese alligator. Considering the high work load of diagnosis and examination for on-site detection and epidemiological investigation, especially when experimental settings are limited, the LAMP assay can be used for the rapid identification of *O. sinensis* infections in the Chinese alligator.

## Supplemental Information

10.7717/peerj.7607/supp-1 Supplemental Information 1 Eight sets of primers of the LAMP reaction for detection of *O. sinensis*. Turbidity was monitored by a Loopamp realtime turbidimeter at 400 nm every 6 s, the curve graph was analyzed every 1 min. NC means double-distilled water.

10.7717/peerj.7607/supp-2 Supplemental Information 2 Different temperatures of the LAMP reaction for detection of *O. sinensis*. Turbidity was monitored by a Loopamp real-time turbidimeter at 400 nm every 6 s, the curve graph was analyzed every 1 min. Eight kinetic curves were generated at various temperatures (60–67 °C, 1 °C intervals) with target pathogens DNA at the level of 0.346 ng per reaction. The curves from 63 to 67 °C showed robust amplification. 65 °C is the first to occur the graphs. NC means double-distilled water.

10.7717/peerj.7607/supp-3 Supplemental Information 3 The specificity of the LAMP reaction for detection of *O. sinensis*. Turbidity was monitored by a Loopamp real-time turbidimeter at 400 nm every 6 s, the curve graph was analyzed every 1 min.

10.7717/peerj.7607/supp-4 Supplemental Information 4 The sensitivity of the LAMP reaction for detection of *O. sinensis*. Turbidity was monitored by a Loopamp real time turbidimeter at 400 nm every 6 s, the curve graph was analyzed every 1 min.

10.7717/peerj.7607/supp-5 Supplemental Information 5 Detection of fecal samples of the LAMP reaction for *O. sinensis*. Turbidity was monitored by a Loopamp real time turbidimeter at 400 nm every 6 s, the curve graph was analyzed every 1 min.

10.7717/peerj.7607/supp-6 Supplemental Information 6 The specificity of the LAMP reaction for detection of *O. sinensis*. A TVR reagent detection method was executed. One µl TVR reagent was added to 25 µl LAMP reaction mixture before the LAMP reaction. Amplification was performed at 65 °C for 50 min.

10.7717/peerj.7607/supp-7 Supplemental Information 7 The specificity of the LAMP reaction for detection of *O. sinensis*. PCR products were analyzed by 2% agarose gel electrophoresis and stained with ethidium bromide.Tubes and lanes: (1) OS (*Ortleppascaris sinensi*); (2) AL(*Ascaris lumbricoides*); (3) AN (*Anisakis* sp.); (4) TSP (*Trichinella spiralis*); (5) CE (*Cucullanus elongatus*); (6) TS (*Taenia solium*); (7) TA (*Taenia asiatica*); (8) LI (*Ligula* sp.); (9) FG (*Fasciola gigantica*); (10) SJ (*Schistosoma japonicum*); (11) NC (double-distilled water).

10.7717/peerj.7607/supp-8 Supplemental Information 8 The sensitivity of the LAMP reaction for detection of *O. sinensis*. The TVR visual color detection was compared using the addition of one µl TVR reagent to 25 µl LAMP reaction mixture before the LAMP reaction

10.7717/peerj.7607/supp-9 Supplemental Information 9 The sensitivity of the LAMP reaction for detection of *O. sinensis*. PCR products were analyzed by 2% agarose gel electrophoresis and stained with ethidium bromide.Tubes and lanes: (1) 34.60 ng/µl; (2) 3.46 ng/µl; (3) 0.346 ng/µl; (4) 34.60 pg/µl; (5) 3.46 pg/µl; (6) 0.346 pg/µl; (7) NC (double-distilled water).

10.7717/peerj.7607/supp-10 Supplemental Information 10 Detection of fecal samples of the LAMP reaction for *O. sinensis*. The TVR visual color detection was compared using the addition of one µl TVR reagent to 25 µl LAMP reaction mixture before the LAMP reaction. Tubes: F1–F6 was the fecal samples of the Chinese alligator infected with the *O. sinensis* and F7–F25 was the fecal samples uninfected Controls; NC means double-distilled water.

## Additional Information and Declarations

### Data Availability

The following information was supplied regarding data availability: The raw data is available in the Supplemental Files.
