# Supplementary figures and images for: Sensitive and rapid detection of Ortleppascaris sinensis (Nematoda: Ascaridoidea) by loop-mediated isothermal amplification
Source: PeerJ. 2019 Sep 6;7:e7607. doi: 10.7717/peerj.7607 (PMC6733237; doi:10.7717/peerj.7607)

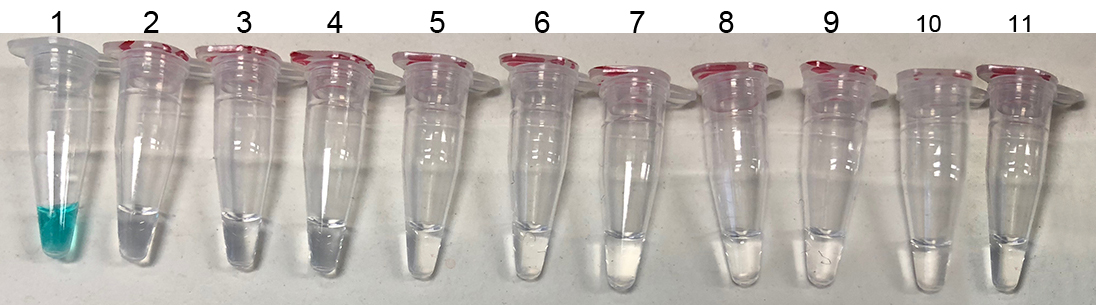

Supplement: Supplemental Information 6 — A TVR reagent detection method was executed. One µl TVR reagent was added to 25 µl LAMP reaction mixture before the LAMP reaction. Amplification was performed at 65 °C for 50 min. [file peerj-07-7607-s006.jpg]

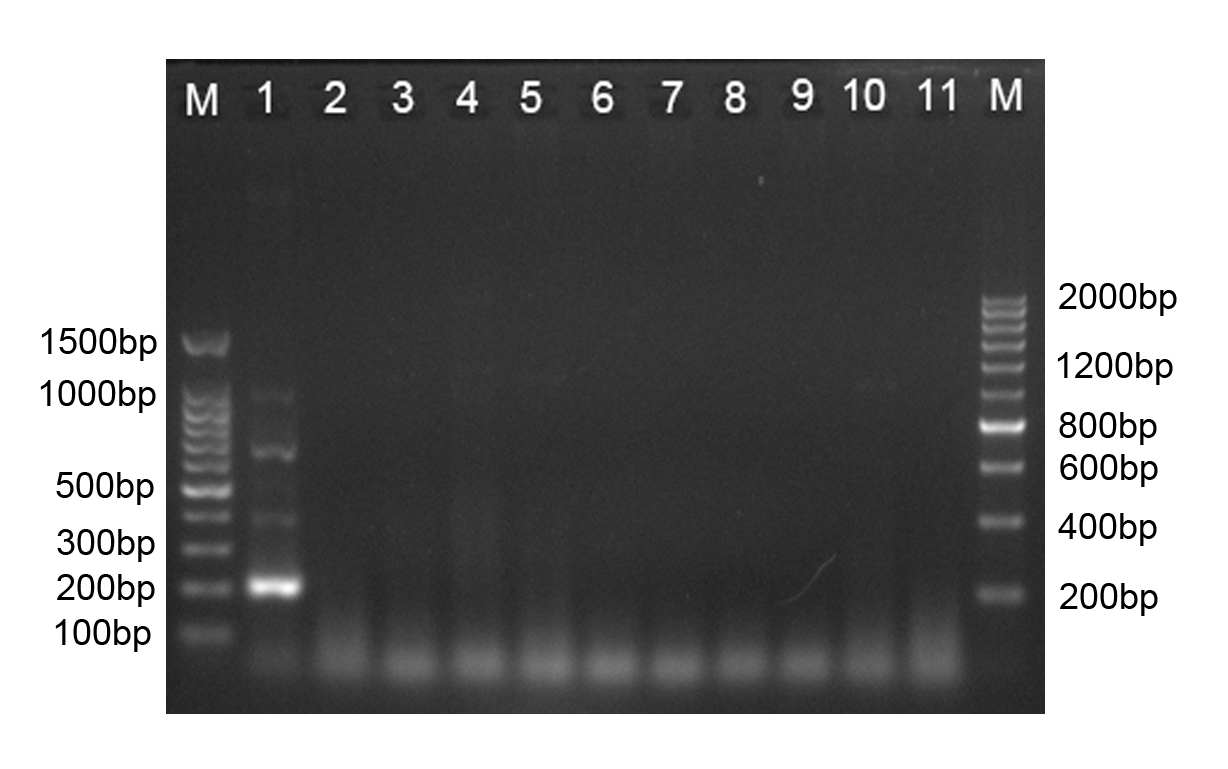

Supplement: Supplemental Information 7 — PCR products were analyzed by 2% agarose gel electrophoresis and stained with ethidium bromide.Tubes and lanes: (1) OS (Ortleppascaris sinensi); (2) AL(Ascaris lumbricoides); (3) AN (Anisakis sp.); (4) TSP (Trichinella spiralis); (5) CE (Cucullanus elongatus); (6) TS (Taenia solium); (7) TA (Taenia asiatica); (8) LI (Ligula sp.); (9) FG (Fasciola gigantica); (10) SJ (Schistosoma japonicum); (11) NC (double-distilled water). [file peerj-07-7607-s007.jpg]

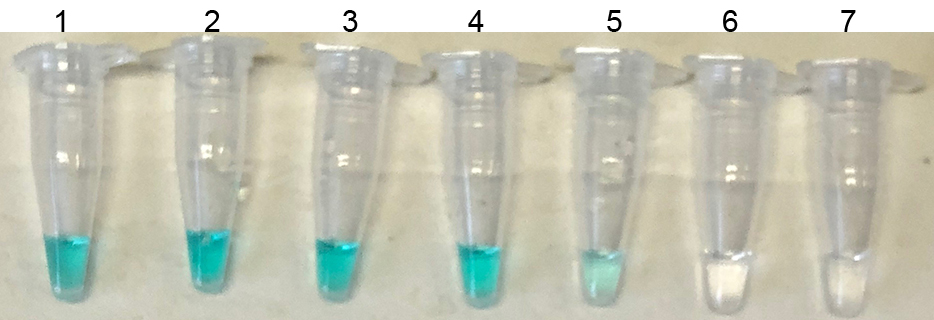

Supplement: Supplemental Information 8 — The TVR visual color detection was compared using the addition of one µl TVR reagent to 25 µl LAMP reaction mixture before the LAMP reaction [file peerj-07-7607-s008.jpg]

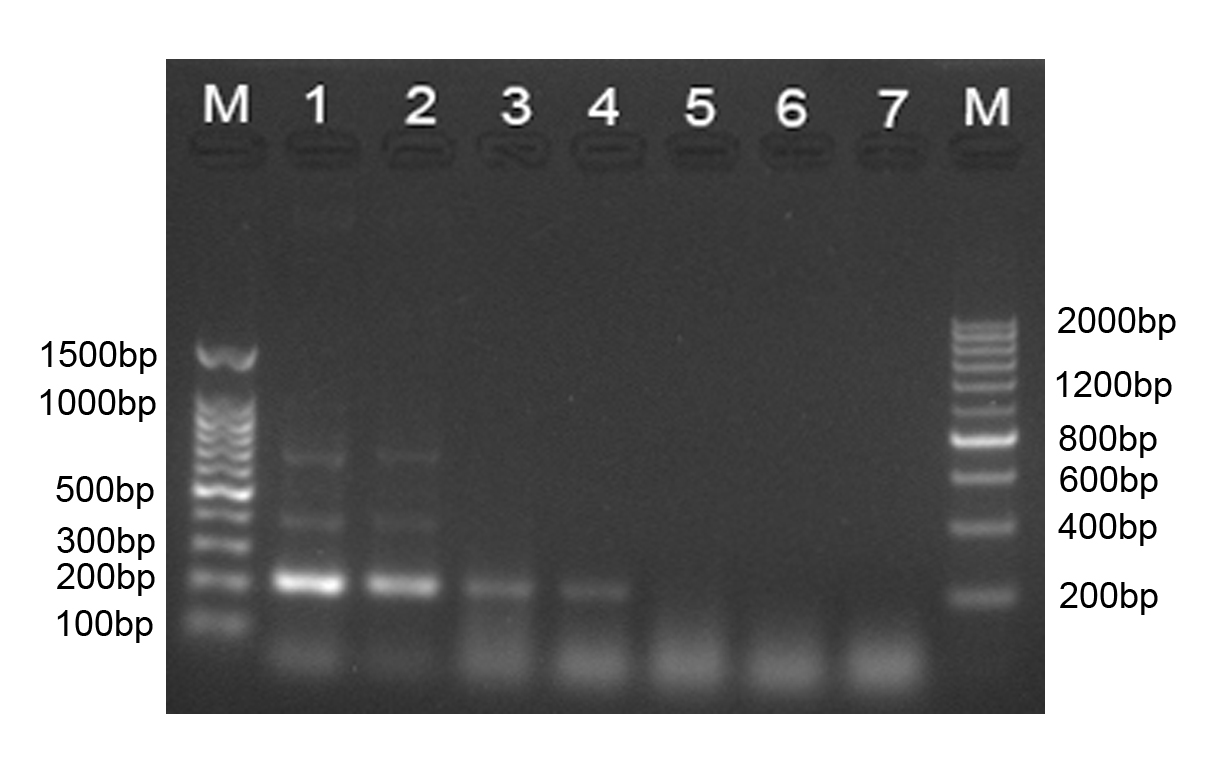

Supplement: Supplemental Information 9 — PCR products were analyzed by 2% agarose gel electrophoresis and stained with ethidium bromide.Tubes and lanes: (1) 34.60 ng/µl; (2) 3.46 ng/µl; (3) 0.346 ng/µl; (4) 34.60 pg/µl; (5) 3.46 pg/µl; (6) 0.346 pg/µl; (7) NC (double-distilled water). [file peerj-07-7607-s009.jpg]

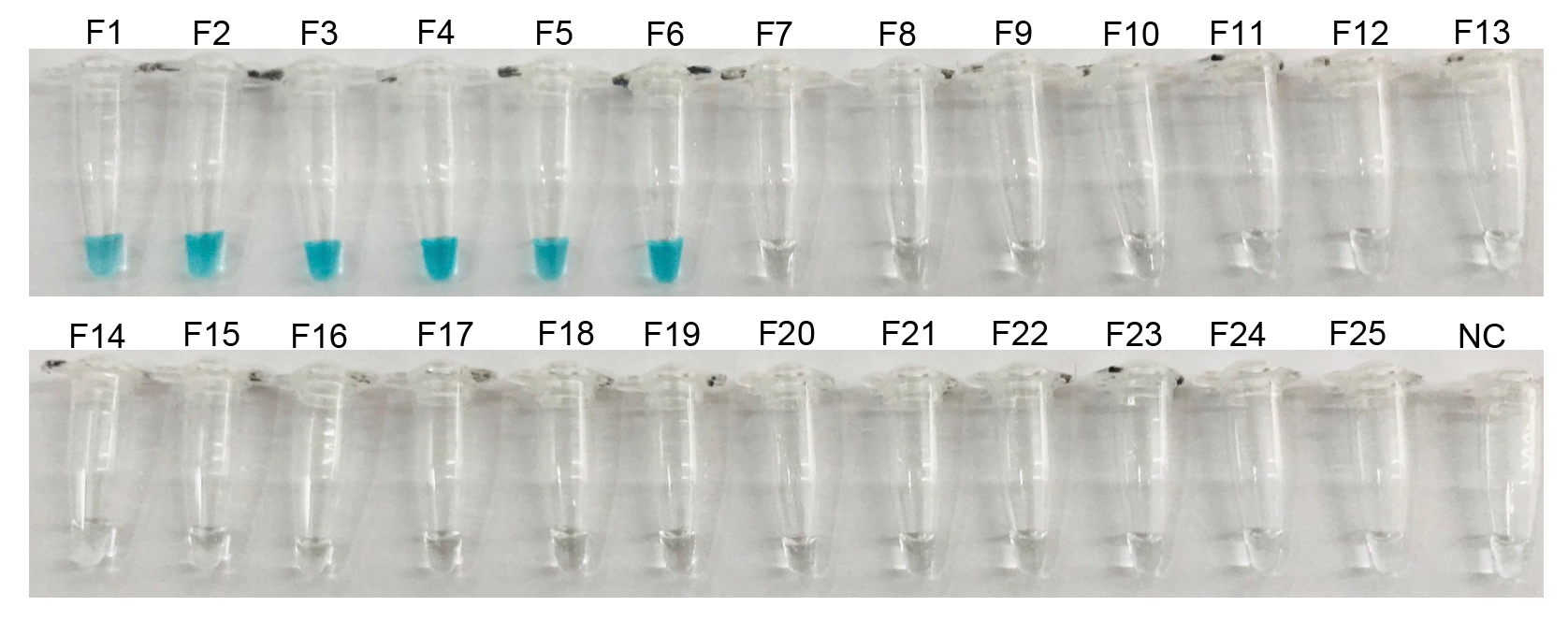

Supplement: Supplemental Information 10 — The TVR visual color detection was compared using the addition of one µl TVR reagent to 25 µl LAMP reaction mixture before the LAMP reaction. Tubes: F1–F6 was the fecal samples of the Chinese alligator infected with the O. sinensis and F7–F25 was the fecal samples uninfected Controls; NC means double-distilled water. [file peerj-07-7607-s010.jpg]
